# Effects of Mn, Zn Additions and Cooling Rate on Mechanical and Corrosion Properties of Al-4.6Mg Casting Alloys

**DOI:** 10.3390/ma13081983

**Published:** 2020-04-24

**Authors:** Chih-Ting Wu, Sheng-Long Lee, Ye-Feng Chen, Hui-Yun Bor, Kung-Hui Liu

**Affiliations:** 1 Department of Vehicle Engineering, Army Academy R.O.C., Jhongli 32092, Taiwan; oc103182@aaroc.edu.tw; 2 Institute of Material Science and Engineering, National Central University, Jhongli 32001, Taiwan; alfred2597@yahoo.com.tw (Y.-F.C.); liukh@ye-fong.com (K.-H.L.); 3 Materials and Electro-Optics Research Division, Chung-Shan Institute of Science and Technology, Longtang 32557, Taiwan; borhy.h12@msa.hinet.net

**Keywords:** Al-4.6Mg alloy, Mn addition, Zn addition, cooling rate, corrosion properties

## Abstract

The mechanical properties of the Al-Mg alloy can be enhanced by adding metallic elements, but a continuous distribution of precipitates at grain boundaries leads to intergranular corrosion during sensitization treatment. In the present work, Mn, Zn additions, water cooling and furnace cooling were executed to investigate their effects on the mechanical and corrosion properties of the Al-4.6Mg alloy. Our results show that adding Mn to Al-4.6Mg alloys may produce grain refinement and dispersion strengthening, increasing tensile strength and hardness. The presence of Mn did not affect the corrosion resistance of Al-Mg alloys. Adding Zn to the Al-4.6Mg alloy increased tensile strength and hardness, but decreased corrosion resistance. Combined, the addition of Mn and Zn to the Al-4.6Mg alloy exhibited the highest tensile strength and hardness, but seriously reduced corrosion resistance. Furnace cooling substituted for water quenching could avoid intergranular corrosion, but slightly decreased the tensile strength and hardness by 7.0% and 6.8%, respectively.

## 1. Introduction

Due to their low weight, Al-Mg alloys have been widely studied over the last twenty years. Liu et al. [1] investigated the effect of element addition on Al-Mg alloy. Krol [2] and Snopinski [3] studied the effect of heat treatment on Al-Mg alloys. Tanski et al. [4,5,6] focused on the effects of equal channel angular pressing on Al-Mg alloys. Different processing has also been carried out on Al-Mg alloys [7,8,9]. A500 alloy is a non-heat-treatable Al-Mg alloy. The strength of this alloy can be improved by solid solution strengthening or dispersion strengthening [10]. Solid solution strengthening is attributed to the presence of Mg solute atoms in the Al matrices. Dispersion strengthening is caused by the presence of dispersed phase in the matrices. Increasing Mg content can enhance the strength of the alloy, but it also leads to intergranular corrosion. This is because the precipitation of β phase (Mg_2_Al_3_) at the grain boundaries occurs more easily at temperature ranges between 50 and 200 °C. The corrosion potential of β phase (−1.24 V) is lower than that of the Al matrix (−0.87 V). Therefore, galvanic corrosion occurs preferentially at the grain boundaries [11,12].

Metallic elements such as Mn and Zn are added to Al-Mg alloys to improve the strength of Al-Mg alloys. Adding Mn to Al-Mg alloys can produce grain refinement and enhance their mechanical properties [13,14,15]. In addition, the precipitation of AlMn phase can further enhance the strength of alloys during homogenization treatment [16,17]. Several studies have reported that combined addition of Mn and Zn leads to the presence of few MnAl_6_ and η (MgZn_2_) particles in the Al-Mg alloy [13,16]. The presence of these particles can produce dispersion strengthening. Although adding Zn to Al-Mg alloys can produce solid solution strengthening, the presence of Zn also impairs their corrosion resistance. Sander et al. [18] reported that increasing Zn content (0.4 wt%–1.5 wt%) causes more severe corrosion damage. Lim [19] and Vuelvas [20] indicated that Zinc in Al-Mg alloys can produce η phase (MgZn_2_), increasing the strength of the alloys. Unfortunately, the corrosion potential of η phase is lower than that of the Al matrices, impairing the corrosion resistance of Al-Mg alloys. In contrast to a reduction in corrosion resistance, Carroll [21] and Unocic [22] reported that adding Zn (0.4 wt%–0.9 wt%) to Al-Mg alloys can improve the corrosion resistance of the alloys. Shuiqing et al. [23] also reported that adding 0.2 wt% Zn to Al-Mg-Si alloy results in the reduction of intergranular corrosion.

The problem of Al-Mg alloy application in marine construction is the precipitation of β phase (Mg_2_Al_3_) along grain boundaries causes intergranular corrosion more easily [11,12]. The studies discussed above indicate that Mn or Zn addition can increase the strength of Al-Mg alloys, but conflicting reports are presented on the corrosion resistance of Al-Mg alloys. The aim of this study is to investigate how Mn and/or Zn additions and cooling rate affect the mechanical properties and corrosion properties of Al-4.6Mg casting alloys.

## 2. Experimental Procedure

### 2.1. Material Preparation

High-purity aluminum ingots (99.99%) were melted in a graphite crucible with a resistance furnace at 730 °C. Suitable amounts of pure Mg (99.99%), pure Zn (99.99%) and Al-75Mn master alloy imported by NEW ZUNI corporation (Taipei, Taiwan) were subsequently added to produce experimental Al-4.6Mg alloys. Table 1 shows the weights of aluminum ingots, pure Mg, Zn and Al-75Mn master alloy for producing 12 kg of each experimental alloy. The melts were degassed with argon for 1 h and then held for 10 min before pouring into a 125 × 100 × 25 mm^3^ permanent mold preheated at 300 °C. The chemical compositions of those alloys were determined by ICP-OES and were listed in Table 2. The Al-Mg, Al-Mg-Mn, Al-Mg-Zn and Al-Mg-Mn-Zn alloys were designated as *alloys A, B, C* and *D*, respectively.

### 2.2. Specimen Preparation

**Figure 1:**
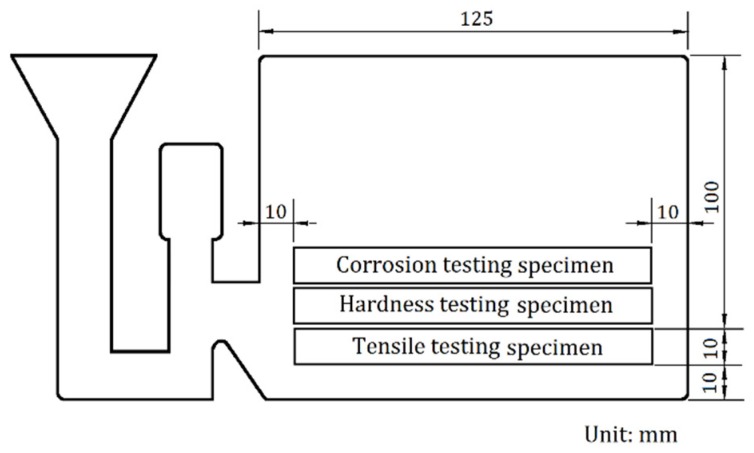
Schematic representation of specimen position in casting.

Sample position: The specimens were cut from casting by using a wire electrical discharge machine. The specimen position of the experimental alloys is shown in Figure 1.

Optical sample: the polished samples were anodized with Barker’s reagent (5 mL HBF_4_ (48%) + 200 mL H_2_O) before immersion in phosphoric acid solution (40 mL H_3_PO_4_ + 60 mL H_2_O). Anodizing condition was 0.2 A/cm^2^ for 60 s at room temperature.

TEM specimen: The specimen was mechanically ground to 50 μm in thickness and then thinned by twin-jet electro-polishing with a solution (30 vol% nitric acid + 70 vol% methanol) at −25 °C and 12 V.

**Figure 2:**
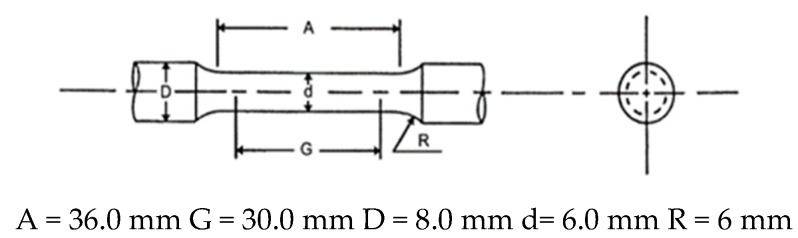
Standard tensile specimen with circular cross section.

Tensile specimen: The dimension of tensile specimen is presented in Figure 2. All tensile specimens were machined by CNC lathe.

Hardness specimen: The dimension of hardness test piece was 105 × 25 × 10 mm.

Corrosion test specimen: The dimension of corrosion test specimen was 50 × 6 × 3 mm.

### 2.3. Equipment

Resistance furnace (Mengli, Hydraulic tilting type, Mengli industrial Co., Ltd. Taiwan); Inductively coupled plasma optical emission spectrometry (Agilent, 725 ICP-OES, Agilent Technologies, Santa Clara, CA, USA); Wire electrical discharge machine (CHMER, MV643S, Ching Hung machinery & electric industrial Co., Ltd., Taiwan); CNC lathe (Focus, FCL-200MC, Focus CNC Co., Ltd., Taiwan); Optical microscope (Olympus, BX60, Olympus corporation, Tokyo, Japan); Scanning electron microscope (JEOL, JEOL-JAM-35CF type, JEOL Ltd., Tokyo, Japan); Transmission electron microscopy (JEOL, JEM-2100, JEOL Ltd., Tokyo, Japan); Electrical conductivity meter (Fisher, SigmaScope SMP10, Fisher Technology, Sindelfingen, Germany); Air circulating furnace (Yokogawa, UP150, Yokogawa Electric Corporation, Tokyo, Japan). Tensile testing (MTS, Universal Testing Machine 10-ton, MTS Systems Corporation, Eden Prairie, MN, USA); Rockwell hardness testing (Matsuzawa, MARK-M2, Matsuzawa Co., Ltd., Akita, Japan).

### 2.4. Heat Treatment

Homogenization treatment: As-cast experimental samples were heated at 480 °C for 8 h in the air circulating furnace.

Water quenching: The samples after homogenization treatment were cooled immediately in the room temperature water.

Furnace cooling: The samples after homogenization treatment were cooled slowly in the air circulating furnace until room temperature.

Sensitization treatment: After water quenching or furnace cooling, the homogenized samples were heated at 160 °C for three days in the air circulating furnace.

### 2.5. Nitric Acid Mass Loss Testing

According to ASTM-G67 [24], the experimental specimens were immersed in the nitric acid solution (70 vol%) at 30 °C for 24 h to obtain the difference in mass before and after nitric acid mass loss testing. The difference in mass could be adopted to detect the susceptibility to intergranular corrosion of the alloys. Materials with mass losses less than 15 mg cm^−2^ were considered resistant to intergranular corrosion; between 15 to 25 mg cm^−2^ metallographic examination was used to determine intergranular corrosion or not; greater than 25 mg cm^−2^ were considered susceptible to intergranular corrosion.

### 2.6. Tensile Testing

Tensile testing was carried out at a strain rate of 1.3 × 10^−3^ s^−1^ according to ASTM B557M-10 [25]. Three tensile specimens were tested, and the average value was calculated.

### 2.7. Hardness Testing

The Rockwell hardness F scale (60-kg load, load time 15 s) was used to measure the hardness. Each hardness specimen was measured ten times and an average value was calculated by excluding the minimum and maximum values.

## 3. Results

### 3.1. Microstructure

**Figure 3:**
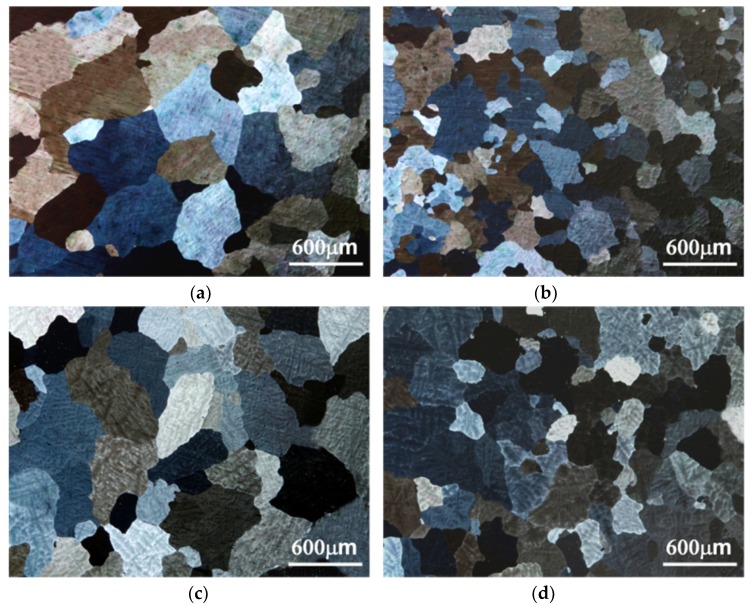
Optical photomicrographs of as-cast (**a**) *alloy A* (Al-Mg alloy), (**b**) *alloy B* (Al-Mg-Mn alloy), (**c**) *alloy C* (Al-Mg-Zn alloy) and (**d**) *alloy D* (Al-Mg-Mn-Zn alloy).

**Figure 4:**
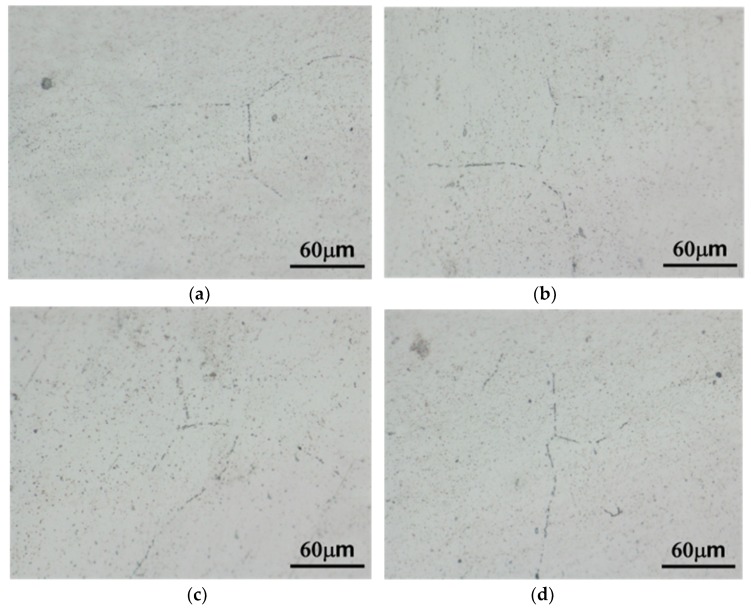
Optical photomicrographs of as-cast (**a**) *alloy A*, (**b**) *alloy B*, (**c**) *alloy C* and (**d**) *alloy D* after immersion in phosphoric acid solution.

**Figure 5:**
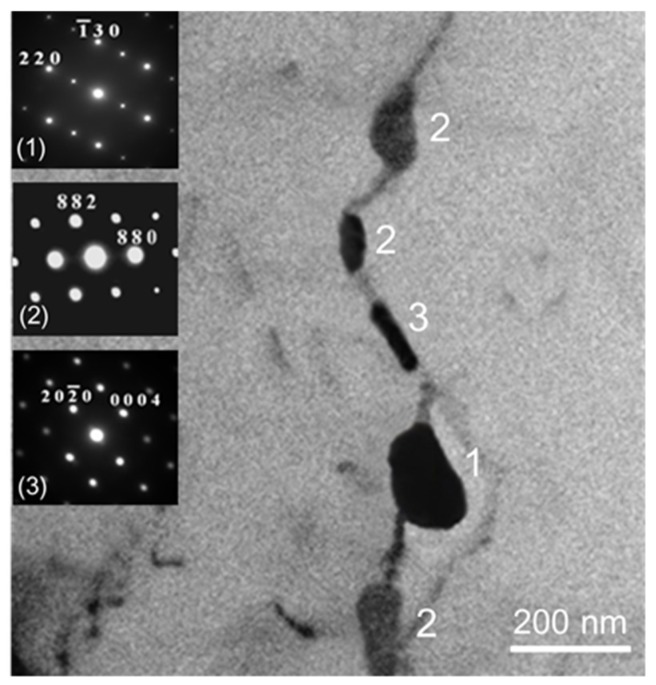
TEM photomicrograph of as-cast *alloy D* (1: MnAl_6_, 2: Mg_2_Al_3_, 3: MgZn_2_).

The grain size of the *alloys B* and *D* was relatively smaller than that of the *alloys A* and *C* in as-cast condition (Figure 3). Few β (Mg_2_Al_3_) at grain boundaries could be found in all the experimental alloys in as-cast condition (Figure 4). Due to Mn and Zn additions, continuous distribution of few β, MnAl_6_ and η phases at the grain boundary could be found in the as-cast *alloy D* by using transmission electron microscopy (Figure 5).

**Figure 6:**
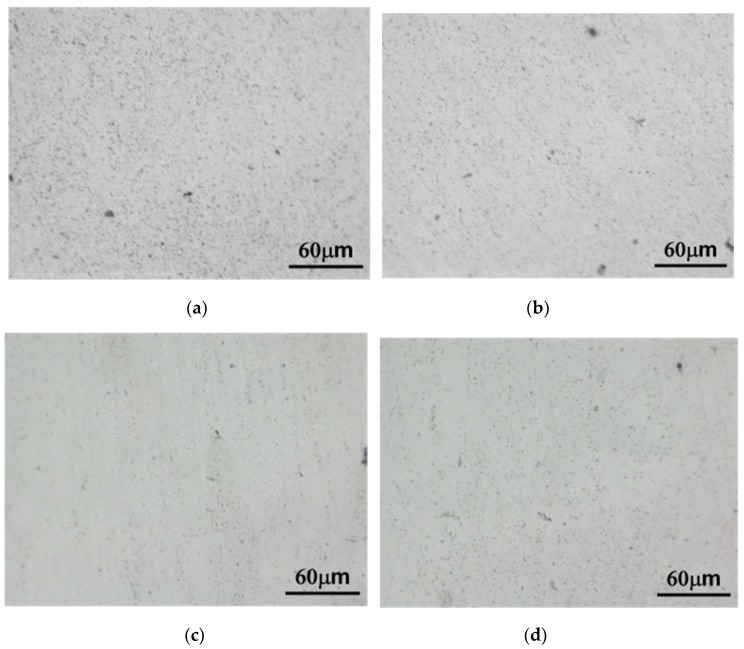
Optical photomicrographs of (**a**) *alloy A*, (**b**) *alloy B*, (**c**) *alloy C* and (**d**) *alloy D* after homogenization treatment.

**Figure 7:**
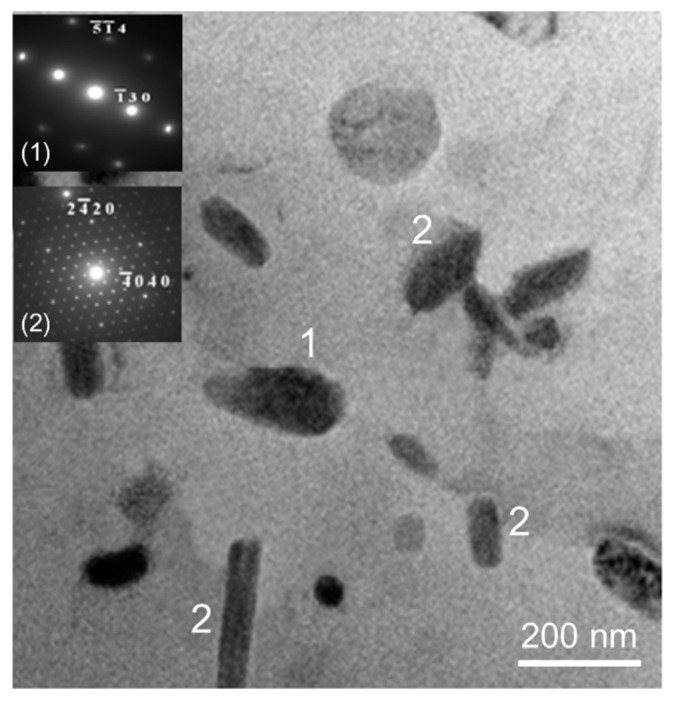
TEM photomicrograph of *alloy D* after homogenization treatment (1: MnAl_6_, 2: MnAl_4_).

The β and η phases at grain boundaries were absent after homogenization treatment and water quenching (Figure 6). Transmission electron microscopy was used to further examine the Al matrices of the *alloy D* after homogenization and quenching. The result indicates that MnAl_6_ and MnAl_4_ could be found in the Al matrices (Figure 7).

**Figure 8:**
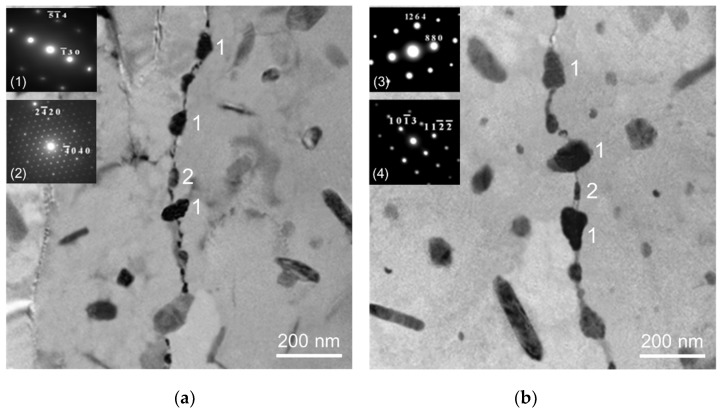
TEM photomicrographs of *alloy D* (**a**) homogenization, water quenching and sensitization for 1 day and (**b**) for 3 days (1: Mg_2_Al_3_, 2: MgZn_2_).

**Figure 9:**
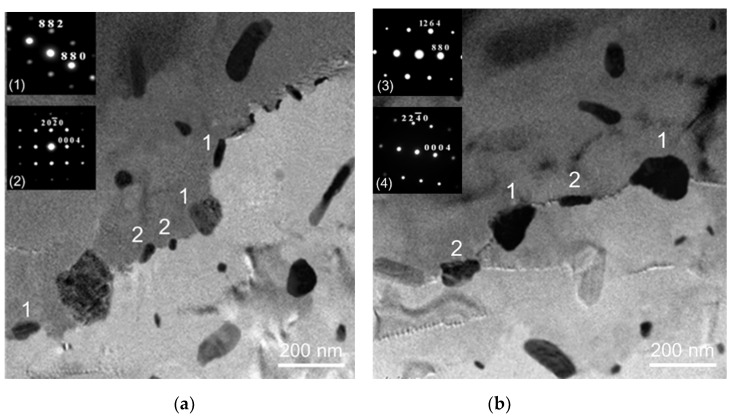
TEM photomicrographs of *alloy D* (**a**) homogenization and furnace cooling (**b**) homogenization, furnace cooling and sensitization (1: Mg_2_Al_3_, 2: MgZn_2_).

Figure 8a shows that the tiny β and η precipitates were present at the grain boundary of *alloy D* (homogenization and water quenching) in the first day of sensitization treatment. After sensitization treatment for 3 days, the coarsening of the β precipitates could be observed and the η precipitates still maintained the original size. Due to the coarsening of β precipitates, the β and η phases almost form a continuous distribution along the grain boundaries in the *alloy D* (Figure 8b). Figure 9a shows the tiny β and η precipitates at grain boundaries in the *alloy D* after homogenization and furnace cooling. The following sensitization led to a discontinuous distribution of β and η precipitates along grain boundaries, as shown in Figure 9b.

### 3.2. Electrical Conductivity Measurements

Table 3 shows the electrical conductivity of all the experimental alloys in different heat treatment conditions. The as-cast *alloy D* (23.34) had the lowest electrical conductivity; the as-cast *alloy A* (33.77) had the highest electrical conductivity. During homogenization, the electrical conductivity of the *alloys A* and *C* only slightly decreased by 0.47% and 0.60%, respectively. In contrast, the *alloys B* and *D* significantly increased their electrical conductivity by 9.36% and 11.44%, respectively. The electrical conductivity of the sensitized alloys was higher than that of the homogenized alloys. The sensitized *alloys C* (2.92%) and *D* (4.27%) with Zn had higher percentage change in electrical conductivity compared to the sensitized *alloys A* (1.76%) and *B* (2.19%) without Zn.

### 3.3. Corrosion Properties

Table 4 shows the results of ASTM G67 nitric acid mass loss test for the experimental alloys in the homogenized and sensitized states. The mass losses of all the homogenized alloys were between 2.7 and 3.1 mg cm^−2^. The mass losses of all the alloys significantly increased after homogenization. In addition, the sensitized *alloys C* and *D* (35.1 and 61.3 mg cm^−2^) were much more susceptible to intergranular corrosion than the sensitized *alloys A* and *B* (15.4 and 15.9 mg cm^−2^). Table 4 also confirms a significant improvement in the corrosion resistance of the furnace-cooled *alloy D* (24.6 mg cm^−2^) compared to the water-quenched *alloy D* (61.3 mg cm^−2^).

**Figure 10:**
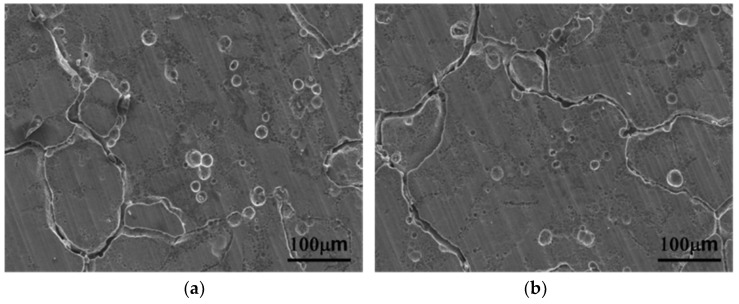

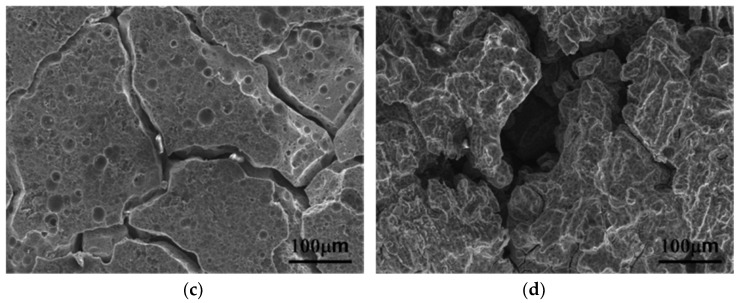
Scanning electron image of corroded surface of (**a**) *alloy A*, (**b**) *alloy B*, (**c**) *alloy C* and (**d**) *alloy D* after sensitization treatment.

**Figure 11:**
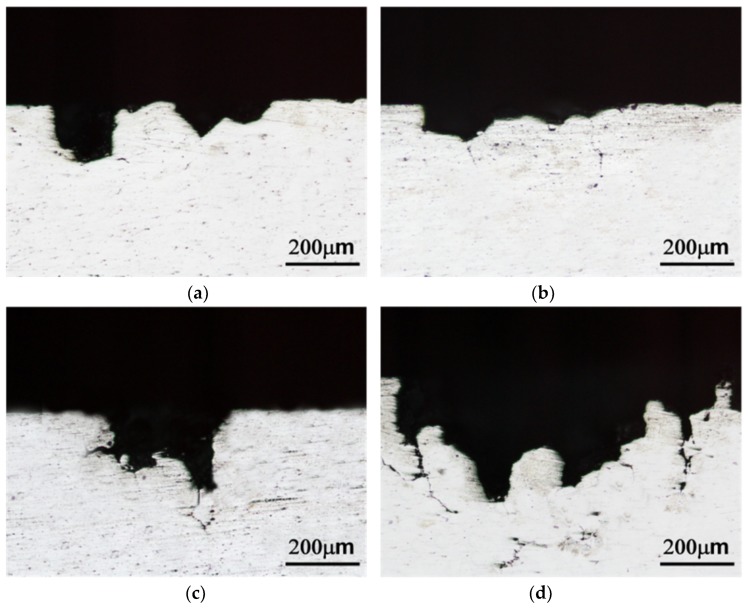
Transverse corroded surface of (**a**) *alloy A*, (**b**) *alloy B*, (**c**) *alloy C* and (**d**) *alloy D* after sensitization treatment.

Figure 10 shows the surface of sensitized alloys after ASTM G67 mass loss test. The pitting corrosion and slight intergranular corrosion could be found in the sensitized *alloys A* and *B*. The sensitized *alloys C* and *D* had significant intergranular corrosion, especially the sensitized *alloy D.* Figure 11 shows the transverse surface of sensitized alloys after ASTM G67 mass loss test. The transverse surface also shows that the corrosion of *alloys C* and *D* was more severe than that of the *alloys A* and *B*.

### 3.4. Mechanical Characteristics

Table 5 shows the hardness and ultimate tensile strength of experimental alloys in different conditions. The hardness and UTS of the as-cast *alloys B* (65.1 HRF, 298.7 MPa) and *D* (66.8 HRF, 309.7 MPa) with Mn were superior to those of the as-cast *alloys A* (51.6 HRF, 252.6 MPa) and *C* (53.9 HRF, 263.2 MPa). Homogenization heat treatments could increase the hardness and UTS of the alloys. In contrast, sensitization treatment decreased of hardness and UTS of the alloys. Compared to the other alloys, the *alloy D* exhibited the highest hardness and tensile strength in each treatment condition.

## 4. Discussion

### 4.1. Microstructure

Adding Mn to Al-4.6Mg casting alloys significantly generated grain refinement. A mechanism of grain refinement is attributed to the presence of nuclei and solute segregation [26,27]. Because the molten alloys cooled through the α + β region, few β (Mg_2_Al_3_) at grain boundaries could be found in all the as-cast alloys [28]. Homogenization treatment led to the dissolution of β (Mg_2_Al_3_) at grain boundaries. Due to their good thermal stability, sensitization treatment could not affect the size and morphology of MnAl_4_ and MnAl_6_ phases in the *alloy D*. In addition, sensitization treatment also caused the precipitation of η phase in the *alloy D*.

The coarsening of β and η precipitates was present along the grain boundary in the *alloy D* (homogenization and water quenching) after sensitization treatment for three days. This is because water quenching after homogenization treatment could produce a large number of supersaturated Mg and Zn solute atoms in the Al matrices [29]. These Mg and Zn solute atoms in the Al matrices could be sufficiently provided to produce continuous precipitation of β and η phases in the following sensitization treatment [30]. In contrast, a discontinuous distribution of the β and η precipitates could be observed in the *alloy D* (homogenization and furnace cooling) after sensitization treatment for three days. This is because the precipitation of β and η phases caused the lack of Mg and Zn solute atoms near the grain boundaries during furnace cooling. Due to the lack of Mg and Zn solute atoms in the Al matrices, the following sensitization could not produce a continuous distribution of β and η precipitates along grain boundaries.

### 4.2. Electrical Conductivity Measurements

The as-cast *alloy D* had the lowest electrical conductivity, mainly because a large number of Mn and Zn solute atoms retained in the Al matrix. The electrical conductivity of the alloy with Mn was much lower than that of the alloy with Zn. This is because the effect of Mn solute atoms (2.94 μΩ·cm per wt%) on electrical resistance is much higher than that of Zn solute atoms (0.094 μΩ·cm per wt%) in the matrices [31].

The dissolution of β and η phases occurred during homogenization and the following quenching inhibited the precipitation of both the phases, increasing the number of solute atoms in the Al matrices. These foreign solute atoms in the Al matrices generated more point defects, lowering electrical conductivity of the alloys [32]. The electrical conductivity of Mn-free alloys decreased slightly, and the Mn-containing alloys significantly increased their electrical conductivity during homogenization. A significant increase in electrical conductivity was attributed to the precipitation of MnAl_4_ in the Mn-containing alloys. A large number of Mn solute atoms were consumed to produce MnAl_4_, increasing the electrical conductivity of the Mn-containing alloys.

The electrical conductivity of the sensitized alloys was higher than that of the homogenized alloys, mainly because the precipitation of β phase lowered the concentration of Mg solute atoms in the Al matrices during sensitization treatment. The Zn-containing alloys had higher percentage change in electrical conductivity compared to the Zn-free alloys in sensitized condition. The precipitation of η phase after sensitization was responsible for their high percentage change in electrical conductivity.

### 4.3. Corrosion Properties

Materials with mass loss less than 15 mg cm^−2^ are considered resistant to intergranular corrosion. In the present work, the mass loss of all the homogenized alloys (about 3 mg cm^−2^) was much lower than 15 mg cm^−2^, indicating that the type of corrosion was resistant to intergranular corrosion. This is because the β and η phases at grain boundary were dissolved during homogenization. Homogenization treatment led to the absence of these β and η phases at grain boundary, which could prevent intergranular corrosion.

Sensitization treatment accelerated the precipitation of β and η phases and caused higher mass loss of the alloys. The Zn-containing alloys were much more susceptible to intergranular corrosion than the Zn-free alloys in the sensitized condition. Zinc played an important role in intergranular corrosion, mainly because the precipitation of β and η phases was a continuous distribution along grain boundaries after sensitization treatment. In addition, the sensitized *alloy D* with more grain boundaries became more susceptible to intergranular corrosion compared to the sensitized *alloy C*.

The presence of severe intergranular corrosion in the sensitized *alloy D* indicated that combined Mn and Zn to Al-4.6Mg alloy was deleterious to corrosion resistance. The examination of corrosive surface of each specimen was in agreement with their mass losses after ASTM G67.

In the present work, cooling rate also affected corrosion resistance. The corrosion resistance of the *alloy D* (homogenization and water quenching) was inferior to that of the *alloy D* (homogenization and furnace cooling). The microstructure examination showed that the former exhibited a continuous distribution of β and η precipitate along the grain boundaries and the latter had a discontinuous distribution. A lot of research indicated that a continuous distribution of the phase along the grain boundaries causes severe intergranular corrosion more easily [33,34]. That is, furnace cooling substituted for water quenching after homogenization could improve intergranular corrosion of the Al-Mg-Mn-Zn alloy.

### 4.4. Mechanical Characteristics

The mechanical properties of the *alloy B* were superior to those of the *alloy A*, mainly because adding Mn to Al-4.6Mg alloys generated grain refinement and solid solution strengthening. Combined Mn and Zn additions to Al-Mg alloy could obtain the highest tensile strength and hardness. This is because β and η phases were dissolved into the Al matrices after homogenization treatment and the following quenching inhibited the precipitation of β and η, increasing the concentration of Mg and Zn solute atoms in the Al matrices of the *alloy D*. These Mg and Zn solute atoms could promote the solid solution strengthening of the *alloy D*. Similarly, due to solid solution strengthening, the hardness and UTS of the as-cast *alloy C* with Zn was superior to those of the as-cast *alloy A*.

The hardness and UTS of all the alloys had a slight decrease after sensitization treatment. This is because the precipitation of β and η phases consumed large numbers of Mg and Zn solute atoms in the Al matrices during sensitization treatment. It is well known that solid solution strengthening depended strongly on the number of solute atoms in the Al matrices. The lower concentration of solute atoms in the Al matrices impaired the effect of solid solution strengthening on the sensitized alloys.

Krol et al. [2] reported that the UTS of Al-5.5Mg alloy is 265.8 MPa. Their heat treatment condition is solution heat treatment at 560 °C for eight hours and aging treatment at 160 °C for four hours. In the present work, heat treatment condition was homogenization treatment at 480 °C for eight hours and sensitization treatment at 160 °C for three days. The UTS of Al-4.6Mg, Al-4.6Mg-Mn, Al-4.6Mg-Zn and Al-4.6Mg-Mn-Zn alloys were 247.6, 302.7, 266.5 and 330.2 MPa, respectively. Due to higher Mg content, the UTS of Al-5.5Mg alloy was higher than that of Al-4.6Mg alloy. Although lower Mg content was added in our experimental alloys, UTS of Al-4.6Mg-Mn and Al-4.6Mg-Mn-Zn alloys was superior to that of Al-5.5 Mg alloy. These results indicated that Mn or combined Mn and Zn additions could increase tensile strength of Al-Mg alloy, especially Al-4.6Mg-Mn-Zn alloys.

## 5. Conclusions

Manganese and/or Zn additions and cooling rate significantly affected the mechanical properties and corrosion properties of Al-4.6Mg casting alloys. The following conclusions are drawn from the experimental results and can be applied in industry:

1. Adding Mn to Al-4.6Mg alloys can produce grain refinement and dispersion strengthening, increasing their tensile strength (329.8 MPa) and hardness (71.3 HRB). The addition of Mn still retains a high resistance to corrosion of the alloy (15.9 mg cm^−2^).
2. The addition of Zn to Al-4.6Mg alloy slightly promotes tensile strength (281.7 MPa) and hardness (57.1 HRB) but noticeably decreases corrosion resistance (35.1 mg cm^−2^).
3. Combined Mn and Zn addition to Al-4.6Mg alloy exhibits the highest tensile strength (336.2 MPa) and hardness (73.4 HRB) but seriously impairs corrosion resistance (61.3 mg cm^−2^).
4. Furnace cooling substituted for water quenching after homogenization treatment can markedly improve corrosion resistance (24.6 mg cm^−2^) and only slightly decreases the tensile strength and hardness of Al-4.6Mg-Mn-Zn alloy by 7.0% and 6.8%, respectively.

## Figures and Tables

**Table 1:** Preparation of materials for each experimental alloy (kg).

Alloy	Mg	Al-75Mn	Zn	Al
*A*	0.56	-	-	11.44
*B*	0.56	0.12	-	11.32
*C*	0.56	-	0.08	11.36
*D*	0.56	0.12	0.08	11.24

**Table 2:** Chemical compositions of experimental alloys (wt%).

Alloy	Mg	Mn	Zn	Al
*A*	4.58	-	-	Rem.
*B*	4.62	0.78	-	Rem.
*C*	4.58	-	0.68	Rem.
*D*	4.61	0.76	0.69	Rem.

**Table 3:** Electrical conductivity of experimental alloys in different conditions.

Alloy	As-Cast C_C_	Homogenization C_W_	Sensitization C_S_	(C_W_ − C_C_)/C_C_ × 100%	(C_S_ − C_W_)/C_W_ × 100%
*A*	33.77 (0.14)	33.61 (0.11)	34.20 (0.12)	−0.47%	1.76%
*B*	23.78 (0.11)	26.07 (0.09)	26.64 (0.10)	9.63%	2.19%
*C*	33.43 (0.08)	33.23 (0.10)	34.10 (0.08)	−0.60%	2.92%
*D*	23.34 (0.08)	26.01 (0.11)	27.12 (0.10)	11.44%	4.27%

Standard deviations are listed in parenthesis.

**Table 4:** ASTM G67 test of experimental alloys in homogenized and sensitized conditions (mg cm^−2^).

Alloy	Homogenization	Sensitization
*A*	3.1 (0.2)	15.4 (0.4)
*B*	2.9 (0.3)	15.9 (0.7)
*C*	2.7 (0.4)	35.1 (1.2)
*D*	3.1 (0.5) 2.9 (0.3)*	61.3 (2.3) 24.6 (1.1) *

*: Homogenization and furnace cooling. Standard deviations are listed in parenthesis.

**Table 5:** Hardness and ultimate tensile strength of experimental alloys in different conditions.

Alloy	As-Cast Hardness UTS (HRB) (MPa)	Homogenization Hardness UTS (HRB) (MPa)	Sensitization Hardness UTS (HRB) (MPa)
*A*	51.6 252.6 (1.1) (4.2)	55.2 263.3 (1.1) (4.5)	50.4 247.6 (0.9) (3.9)
*B*	65.1 298.7 (1.1) (3.2)	71.3 329.8 (1.3) (4.2)	66.9 302.7 (1.2) (3.2)
*C*	53.9 263.2 (1.0) (4.4)	57.1 281.7 (1.0) (2.1)	55.4 266.5 (1.3) (4.2)
*D*	66.8 309.7 (1.2) (2.5)	73.4 336.2 (1.1) (4.0) 68.4 * 312.7* (1.3) (3.1)	71.6 330.2 (1.2) (3.6) 67.2 * 305.6 * (1.4) (1.1)

*: Homogenization and furnace cooling. Standard deviations are listed in parenthesis.
